# Independent predictors of physical health in community-dwelling patients with coronary heart disease in Singapore

**DOI:** 10.1186/s12955-016-0514-7

**Published:** 2016-07-28

**Authors:** Wenru Wang, Ying Jiang, Chi-Hang Lee

**Affiliations:** 1 Alice Lee Centre for Nursing Studies, Yong Loo Lin School of Medicine, National University of Singapore, Level 2, Clinical Research Centre, Block MD 11, 10 Medical Drive, Singapore, 117597 Singapore; 2 Yong Loo Lin School of Medicine, National University of Singapore, Singapore, Singapore

**Keywords:** Coronary heart disease, Singaporean patients, Independent predictors, Physical health

## Abstract

**Background:**

Patients with coronary heart disease (CHD) experienced poor physical health which was found to be associated with higher hospital readmission rates and increased mortality. The study aimed to identify the independent predictors of physical health in Singaporean patients with CHD.

**Methods:**

A consecutive sample of 129 patients with CHD was recruited from the medical heart clinic of a tertiary public hospital in Singapore. A set of questionnaires including the Short Form 12-item health survey version 2, Perceived Stress Scale, Hospital Anxiety and Depression Scale, and Cardiac Self-Efficacy Scale were used to measure the study outcomes. The patients’ socio-demographic and clinical data were also collected.

**Results:**

A multivariate linear regression analysis indicated that depression (B = −0.766, *p* < 0.05) and self-efficacy for maintaining function (B = 2.351, *p* < 0.05) remained significant while the other variables were adjusted and identified as the independent predictors of physical health in Singaporean patients with CHD.

**Conclusions:**

This study has shed some light on the key factors influencing the physical health of Singaporean patients with CHD. The finding suggests tailored interventions that target mitigating a patient’s depression and promote self-efficacy for maintaining function may be helpful in improving patients’ physical health and quality of life.

## Background

Coronary heart disease (CHD) is one of the leading causes of death and disability among adults worldwide, even in Singapore [1]. It has been well documented that patients with CHD experience poor health-related quality of life (HRQoL) [2, 3]. The HRQoL is a complex and multidimensional construct including physiological, emotional, and psychosocial components [4]. In particular, the physical health component of HRQoL was an important outcome measure for patients with CHD [5]. Poor physical health was found to be associated with higher hospital readmission rates and increased mortality [5]. Numerous studies have identified the determinates of physical health in patients with CHD, and many factors have been reported to contribute to the poorer physical health of patients with CHD. These include: (1) socio-demographic factors: age, gender, race, and social economic status (including education, income, and occupation) [6–8]; (2) clinical factors: cardiac-related comorbidities such as hypertension and diabetes, type of diagnosis, revascularization therapies, and severity of the disease [7, 9]; (3) psychosocial factors: anxiety and depression, social support, and stress [4, 10]. However, our recent systematic literature review indicated that there is limited knowledge of the Asian CHD population with regards to their perceptions of the HRQoL and the influencing factors [1]. A deeper understanding of the independent predictors of physical health for Singaporean patients with CHD may help health professionals to develop effective interventions to address the key issues.

We adapted the Revised Wilson and Cleary model of HRQoL as the conceptual framework in guiding this study [11]. The Revised Wilson and Cleary model of HRQoL combined the paradigms of biomedical and social science, and posited HRQoL was influenced by both individual and environmental characteristics such as clinical, psychological, social and demographic status. We recently conducted a randomized controlled trial for community-dwelling patients with CHD in Singapore (Trial Registration Number: ISRCTN15839687). This report is the analysis of the baseline data of the trial, and the aim of this short report is to identify the independent predictors of physical health of Singaporean patients with CHD.

## Methods

### Participants

The study was conducted in the medical heart clinic of a tertiary public hospital in Singapore. The target population of the study was community-dwelling patients who had been diagnosed with CHD and were living in Singapore. A consecutive sample of 129 patients were recruited from April 2015 to August 2015. The patients were invited to participate in the study when they visited the clinic for a regular follow-up consult or medical check-up. Eligible participants were those who: (1) were clinically diagnosed with CHD, including angina pectoris and myocardial infarction; (2) were outpatients and living at home; (3) were 21 years old and above; and (4) were able to converse in English or Chinese. Those patients who suffered complications such as uncontrolled arrhythmias, were on active cancer treatments, or had a known history of neurological or psychological disorder that would impair their normal mental functioning were excluded.

Based on Cohen’s [12] recommendation, when using multiple linear regression analysis with an approximate of ten independent variables [4, 6–10], a minimum of 120 participants would be needed to achieve the median effect size and 80 % statistical power at significant level of 0.05 [12].

### Outcome measurements

#### Social-demographic and clinical data

The social-demographic data of the participants, including their age, gender, marital status, educational level, employment status, monthly household income, and type of housing, were obtained. Medical history including the length of CHD diagnosis (months), CHD family history, co-morbidities (diabetes, high cholesterol, and hypertension), smoking status, percutaneous transluminal coronary angioplasty/stent therapy, coronary artery bypass grafting, medications, and clinical data including lipid profile, fasting blood glucose, body mass index (BMI), and blood pressure were retrieved from the participants’ latest available medical records. Dyslipidaemia was defined as having one or more of the three abnormal lipid levels: (1) triglyceride > 2.26 mmol/l, (2) LDL cholesterol >3.3 mmol/l, and (3) HDL cholesterol <1.03 (for men) and <1.30 mmol/l (for women) [5]. Obesity was defined as body mass index (BMI) >30 kg/m^2^ [5].

#### Short form 12-item health survey version 2

The overall HRQoL was measured by the Short Form 12-item health survey version 2 (SF-12v2). The SF-12v2 consists of 12 items which measure the physical health domain (physical component summary (PCS)) and mental health domain (mental component summary (MCS)) of the participants’ quality of life [9]. A higher score indicates a better HRQoL. The SF-12v2 has good internal consistency with Cronbach’s alphas of 0.87 and 0.82 for the PCS and MCS respectively [9].

#### Perceived stress scale

The Perceived Stress Scale (PSS) is used to measure the participants’ perceived level of stress [13]. It consists of ten items used to measure the degree of how unpredictable, uncontrollable, and overloaded the respondents find their lives are. A higher score indicates a higher perceived level of stress. The PSS has good internal consistency with Cronbach’s alpha of 0.89 for the overall scale [13].

#### Hospital anxiety and depression scale

The Hospital Anxiety and Depression Scale (HADS) is a widely used instrument to assess for the presence of anxiety or depression in patients who are physically ill [14]. It consists of 14 items with seven of the items measuring the anxiety level and another seven items measuring the depression level. Each item was scored from zero to three, giving a range of 0 to 21 for either anxiety or depression subscale. A higher score indicated higher anxiety or depression level. It has good internal consistency with a Cronbach’s alpha of 0.85 for the anxiety subscale, and 0.79 for the depression subscale [15].

#### Cardiac self-efficacy scale

Self-efficacy was measured by the Cardiac Self-Efficacy Scale (CSS). It is a self-reported inventory to assess the participants’ level of confidence on knowing or acting on 16 statements. The scale consists of two factors: self-efficacy for controlling symptoms (CSS-CS) and self-efficacy for maintaining function (CSS-MF). The internal consistency was reported to be acceptable, with Cronbach’s alphas of 0.90 and 0.87 for CSS-CS and CSS-MF respectively [16]. The convergent and discriminant validity were reported to be good as well [16].

### Data collection procedure

Ethical approval was obtained from the National Health Group Domain Specific Review Board (reference number: 2013/00727) before the start of the study. A trained research nurse approached all potential study participants for recruitment. Informed consent was obtained from the participants before data collection. Sufficient time and privacy were given to the participants to complete the survey. On average, each participant took about 20 min to complete all of the questionnaires.

### Data analysis

Data were analyzed using the IBM SPSS version 22.0 (SPSS Inc. Chicago IL, USA). Descriptive statistics were used to summarize the participants’ socio-demographic data, clinical characteristics, anxiety and depression level, perceived stress level, health quality of life, and cardiac self-efficacy. Physical health was measured by the PCS of SF-12v2. Univariate regression analyses were performed to identify the potential associating factors of the PCS. Those variables with a *p*-value of less than 0.25 [17] in univariate regression analyses were chosen as potential independent predictors for the multivariate linear regression. Data were verified for assumptions required for regression. Unstandardized residual was normally distributed, with the value of Durbin-Watson diagnostic test of 2.047. The significant value was set at *p* < 0.05.

## Results

### Demographic and clinical characteristics

Out of the 328 patients that were screened, 166 were approached and 129 patients were recruited and completed the questionnaires (response rate: 77.7 %). The socio-demographic and clinical characteristics of the participants are summarized in Table 1. The age of the participants ranged from 35 to 80 years old (mean = 60.9, SD = 8.8). The majority of participants were male (89.1 %) and have attained secondary and above educational qualification (73.6 %). More than half of the participants (67.4 %) were working full-time or part-time, with 53.5 % earning a monthly household income of equal to or less than SGD3,000 (1SGD = 0.74USD), much lower than the median household income (SGD8,290) in Singapore [18]. In terms of clinical characteristics, the length of diagnosis of CHD ranged from 1 month to 30 years, all participants (100 %) had a history of high cholesterol, 83.7 % had hypertension, and close to half (44.2 %) of them had Type 2 Diabetes. 57.4 % of the participants were ex-smokers. The majority of the participants were on medications of antiplatelets (95.3 %), beta blockers (69 %), and lipid-lowering drugs (94.6 %).

**Table 1:** Socio-demographic and clinical characteristics of the study sample (*n* = 129)

Characteristic	Sample (*n* = 129)
Gender (n, %)	
Male	115 (89.1)
Female	14 (10.9)
Age (years)	60.9 ± 8.8
Marital status (n, %)	
Married	106 (82.2)
Single/Divorced/Windowed/Separated	23 (17.8)
Highest education level (n, %)	
No formal education	8 (6.2)
Primary education	26 (20.2)
Secondary education	60 (46.5)
Tertiary education and above	35 (27.1)
Employment status (n, %)	
Full-time/Part-time working	87 (67.4)
Retired/Not working	42 (32.6)
Monthly household income (SGD)	
≤ 1000	23 (17.8)
1001–3000	46 (35.7)
3001–5000	30 (23.3)
> 5000	29 (22.5)
Type of housing (n, %)	
Government house/HDB flat	122 (94.6)
Private house	7 (5.4)
Clinical characteristics	
Length of CHD diagnosis (months)	36.0 (1.0–360.0)
CHD family history (n, %)	55 (42.6)
Diabetes	57 (44.2)
High cholesterol	129 (100)
Hypertension	108 (83.7)
Ex-smoker	74 (57.4)
Current smoker	26 (20.2)
Alcohol	30 (23.3)
PTCA/Stent therapy	89 (69.0)
CABG therapy	31 (24.0)
Medication	
Antiplatelets	123 (95.3)
Nitrates	34 (26.4)
Beta-blockers	89 (69.0)
ACE inhibitors	58 (45.0)
Calcium antagonists	28 (21.7)
Lipid-lowering drugs	122 (94.6)
Others	120 (93.0)
Physiological data	
Triglycerides (mmol/L)	1.38 (0.52–16.01)
Total cholesterol (mmol/L)	4.06 (2.45–10.52)
Low-density lipoprotein (mmol/L)	2.18 (0.53–6.07)
High-density lipoprotein (mmol/L)	1.11 ± 0.24
Fasting blood glucose (mmol/L)	5.75 (4.00–22.50)
Systolic blood pressure (mmHg)	135.90 ± 23.51
Diastolic blood pressure (mmHg)	74.22 ± 11.41
BMI	26.70 ± 5.04

1SGD = 0.74USD

*PTCA* percutaneous transluminal coronary angioplasty, *CABG* coronary artery bypass grafting, *BMI* body mass index

### Anxiety and depression level, perceived stress, cardiac self-efficacy, and HRQoL

The summaries’ scores of the study variables are presented in Table 2. Among all the participants, 16 participants (12.4 %) experienced higher levels of anxiety, while 12 participants (9.3 %) experienced higher levels of depression using the cut-off of 8 points for both the anxiety and depression scales [14]. Anxiety (*r* = −0.36, *p* < 0.05), depression (*r* = −0.51, *p* < 0.05), and perceived stress (*r* = −0.43, *P* < 0.05) were found to be significantly negatively correlated with the PCS. By contrast, CSS-CS (*r* = 0.35, *P* < 0.05) and CSS-MF (*r* = 0.54, *p* < 0.05) were significantly positively correlated with the PCS.

**Table 2:** Mean scores and correlations among hospital anxiety and depression scale, perceived stress scale, cardiac self-efficacy scale and health-related quality of life

Variables	Mean (SD)	Pearson’s correlation	
		Physical component summary	Mental component summary
HADS			
HADS_Anxiety	3.36 (3.60)	−0.36*	−0.57*
HADS_Depression	3.05 (2.95)	−0.51*	−0.56*
Perceived stress scale	11.33 (7.11)	−0.43*	−0.57*
Cardiac self-efficacy scale			
Controlling symptoms	3.17 (0.73)	0.35*	0.25*
Maintaining functioning	3.03 (0.95)	0.54*	0.42*
SF-12v 2			
Physical component summary	46.35 (8.71)	1	0.34*
Mental component summary	50.30 (9.22)	0.34*	1

*HADS* Hospital Anxiety and Depression Scale, *SF-12 V2* Short Form 12-item health survey version 2.

*p < 0.05

### The independent predictors of physical health

Table 3 presents the unadjusted and adjusted predictors for the PCS. The univariate regression analysis results showed that monthly household income (*p* < 0.001), type of housing (*p* < 0.05), history of diabetes (*p* < 0.05), perceived stress level (*p* < 0.001), anxiety level (*p* < 0.001), depression level (*p* < 0.001), CSS-CS (*p* < 0.001), and CSS-MF (*p* < 0.001) were significant predictors for the PCS. the multivariate linear regression analysis showed that only the depression level (B = −0.766, *p* < 0.05) and CSS-MF (B = 2.351, *p* < 0.05) remained significant while the other variables were adjusted and identified as the independent predictors of the PCS. In addition, CHD family history became significant (B = 2.967, *p* < 0.05) after adjusting other variables. The predicted model was statistically significant (F = 5.183, *p* < 0.001), and the three predictors (i.e., depression, CSS-MF, and CHD family history) accounted for 42 % of the variance.

**Table 3:** Unadjusted and adjusted predictors for the physical component summary of SF-12v2

Variables	Univariate analysis		Multivariable analysis	
	B	*p*-value	B	*p*-value
Gender	0.107	0.966		
Age	0.038	0.665		
Marital status	2.854	0.155*	1.890	0.296
Highest education level	0.074	0.966		
Employment status	−0.907	0.581		
Monthly household income	4.954	<0.001**	−0.013	0.993
Type of housing	6.861	0.042**	2.600	0.435
Length of CHD diagnosis (months)	−0.022	0.057*	0.007	0.591
CHD family history	2.194	0.158*	2.967	0.044**
Diabetes	3.256	0.034**	0.377	0.802
Hypertension	−1.707	0.414		
Ex-smoker	2.394	0.123*	1.835	0.199
Current smoker	0.727	0.608		
Alcohol	1.768	0.332		
PTCA/stent therapy	0.879	0.598		
CABG therapy	−1.868	0.300		
Dyslipidaemia	−3.142	0.051*	−1.464	0.320
Fasting blood glucose (>6.1 mmol/L)	−1.245	0.526		
BMI (>30)	0.067	0.975		
PSS	−0.530	<0.001**	−0.110	0.439
HADS_Anxiety	−0.881	<0.001**	0.028	0.926
HADS_Depression	−1.514	<0.001**	−0.766	0.023**
CSS-CS	4.173	<0.001**	1.423	0.223
CSS-MF	4.987	<0.001**	2.351	0.019**

Reference group: Male, Single/Divorced/Windowed/Separated, Primary or below education, Full-time/part-time working, monthly household income S$3000 (USD 2217) or below, Government house/HDB flat, has CHD family history, has diabetes, has hypertension, was an ex-smoker, currently smoking, has alcohol consumption, has PTCA/stent therapy, has CABG therapy, no dyslipidaemia, fasting blood glucose > 6.1 mmol/L, BMI less than 30

*CHD* coronary heart disease, *PTCA* percutaneous transluminal coronary angioplasty, *CABG* coronary artery bypass grafting, *BMI* body mass index, *PSS* Perceived Stress Scale, *HADS* Hospital Anxiety and Depression Scale, *CSS_CS* cardiac self-efficacy for control ling symptoms, *CSS-MF* cardiac self-efficacy for maintain function

*P < 0.25, **P < 0.05

## Discussion

HRQoL is the subjective evaluation of the impact of an illness and its related treatment on patient’s life [19]. It is an important outcome that has been widely used to measure treatment satisfaction and efficacy [3]. Past evidence has shown that patients with CHD tended to have a poor HRQoL compared to the healthy population [20]. Results from the current study revealed that the participants’ HRQoL in the current sample is similar to the previous studies conducted in Hong Kong and mainland China [4, 5]. However, when compared to the recent study done locally [1], the participants in the current sample reported a poorer HRQoL in both physical and mental aspects. In addition, there was a higher percentage who reported high levels of anxiety or depression.

Anxiety and depression are the two psychological factors that are commonly identified as strong predictors to a poorer HRQoL among patients with CHD [20–23]. Both were found to be associated with recurrent heart attacks and risks of death [24–26]. In the current study, anxiety, depression, and perceived stress were found to be negatively correlated to physical health. Such finding is not surprising, as patients with CHD often have symptoms like chest pain and breathlessness which give rise to the sense of impending doom and aggravate patients’ fear of another heart attack. Because of the fear and the sense of uncertainty, many patients have altered their lifestyle with the goal of preventing the attack [27, 28] and become apprehensive in returning to their normal routines [28]. On the other hand, while grappling with new changes, the inability to fulfil one’s role (social, familial, or professional) may further distort their self-images and contribute to their mounting anxiety [29]. In the current sample, the majority of the participants were from a lower social economic class, with 67.4 % still working and 53.5 % earning a monthly household income of equal to or less than SGD3,000, much lower than the median household income (SGD8,290) in Singapore [18]. Barbareschi and Sanderman [30] argued that a low-SES individual possessed limited resources to promote wellbeing and had higher psychosocial stress. Many of our participants faced the stress to make ends meet, thus engaging in secondary prevention behaviours may become less of a priority. Consequently, the reduced engagement in health behaviours results in an accelerated disease progression and a poorer HRQoL.

By contrast, CSS-CS and CSS-MF were found to be positively correlated with PCS in the current study. These findings are consistent with evidences from the literature supporting the association between self-efficacy and physical health [31–33]. Low cardiac self-efficacy was found to be associated with a poorer health status even after it was adjusted for disease severity and depressive symptoms [34]. LaPier and Cleary [33] reported that older patients reported higher levels of cardiac self-efficacy, suggesting that life experiences and coping skills may help increase a patient’s confidence in self-management behaviours. Furthermore, while lower cardiac self-efficacy was associated with poorer physical function and less physical activities, patients with low physical function may actually have the greatest improvements in self-efficacy after engaging in physical activities [33]. These findings suggest that self-efficacy is an important and perhaps more easily modified factor that influences the physical health component of patients with CHD.

In this study, depression and CSS-MF were identified as the independent predictors for the PCS. In addition, CHD family history was found to be a significant predictor for the PCS after adjusting other variables. These three predictors accounted for 42 % of the variance. The results suggested that a high level of depression predicted poorer physical health, while a higher level of CSS-MF predicted better physical health. Interestingly, after adjusting other variable, no family history of CHD predicted better physical health. Depression has been consistently identified as the factor that has a major impact on the HRQoL of CHD patients worldwide, including in Asian patients [1, 4, 5, 10]. Depression impairs the patients’ engagement in secondary prevention behaviours such as exercise and medication adherence, which are crucial in delaying disease progression [2]. Lee et al. [5] added that many individual risk factors (modifiable and non-modifiable) that influence the physical component of the HRQoL of CHD patients were mediated by psychological distress. Therefore, providing interventions to mitigate the psychological distress for this group of patients may be more helpful and effective in enhancing treatment outcome [5]. Similarly, CSS-MF could be another modifiable factor that is important in maintaining patients’ physical health. Like depression, CSS-MF probably affects a patient’s physical health through its association with an individual’s engagement in secondary preventive behaviours. Oka and Gortner [35] noted that self-efficacy was a strong mediating factor that influences patients’ activity levels, and that it was a better predictor of physical activity than peak oxygen consumption. Many studies have also reported that there was a strong connection between self-efficacy and exercise behaviour, and functional status and quality of life in patients with CHD [36, 37]. Lastly, family history of CHD became one of the significant predictors after adjusting other variables. This finding gave us a heads-up that we might have missed out on significant findings if we only included the significant univariates into the multivariate analysis. Nevertheless, the presence of family history of CHD may indicate a genetic predisposition that links to poorer physical health.

## Limitation and conclusion

Several factors must be considered when interpreting the results of this study. First, this was a cross-sectional analysis of the baseline data from the randomised controlled trial study, therefore caution must be exercised when extrapolating these results to CHD patients in other settings. Second, the use of self-reported questionnaires may result in social desirability responses. Third, despite we had recruited a total of 129 participants, it is still possible that current study may be under power as there were more than ten independent variables in the final multivariate linear regression analysis. Nevertheless, the current study has shed some light on the key factors influencing the physical health component of the HRQoL in patients with CHD in Singapore. Healthcare practitioners may have started to recognize that patients with a poorer physical health component in their HRQoL not only have a poorer overall quality of life, but also impaired physical function. In this sample, we identified depression and CSS-MF as the independent predictors for the physical health component of the HRQoL. This finding suggests tailored interventions that target mitigating one’s psychological distress and promote self-efficacy may be helpful in improving patients’ physical health and quality of life. Healthcare professionals may incorporate routine screenings for symptoms of depression and provide early intervention. This study also emphasizes the importance of going beyond brief discharge instructions and equipping patient coping skills in dealing with life changes arising from CHD. These measures will better prepare patients to deal with the challenges related to CHD, improve self-efficacy and eventually improve HRQoL.

## Abbreviations

BMI, body mass index; CHD, coronary heart disease; CSS, cardiac self-efficacy scale; CSS-CS, self-efficacy for controlling symptoms; CSS-MF, self-efficacy for maintaining function; HADS, hospital anxiety and depression scale; HRQoL, health-related quality of life; MCS, mental component summary; PCS, physical component summary; PSS, perceived stress scale; SF-12v2, short form 12-item health survey version 2
